# 3D Printing of Elastomeric Bioinspired Complex Adhesive Microstructures

**DOI:** 10.1002/adma.202103826

**Published:** 2021-08-15

**Authors:** Cem Balda Dayan, Sungwoo Chun, Nagaraj Krishna‐Subbaiah, Dirk‐Michael Drotlef, Mukrime Birgul Akolpoglu, Metin Sitti

**Affiliations:** ^1^ Physical Intelligence Department Max Planck Institute for Intelligent Systems 70569 Stuttgart Germany; ^2^ Department of Electronics and Information Engineering Korea University Sejong 30019 Republic of Korea; ^3^ Institute for Biomedical Engineering ETH Zürich Zürich 8092 Switzerland; ^4^ School of Medicine and College of Engineering Koç University Istanbul 34450 Turkey

**Keywords:** bioinspired microstructures, gecko‐inspired adhesives, liquid super‐repellency, reversible adhesion, two‐photon polymerization

## Abstract

Bioinspired elastomeric structural adhesives can provide reversible and controllable adhesion on dry/wet and synthetic/biological surfaces for a broad range of commercial applications. Shape complexity and performance of the existing structural adhesives are limited by the used specific fabrication technique, such as molding. To overcome these limitations by proposing complex 3D microstructured adhesive designs, a 3D elastomeric microstructure fabrication approach is implemented using two‐photon‐polymerization‐based 3D printing. A custom aliphatic urethane‐acrylate‐based elastomer is used as the 3D printing material. Two designs are demonstrated with two combined biological inspirations to show the advanced capabilities enabled by the proposed fabrication approach and custom elastomer. The first design focuses on springtail‐ and gecko‐inspired hybrid microfiber adhesive, which has the multifunctionalities of side‐surface liquid super‐repellency, top‐surface liquid super‐repellency, and strong reversible adhesion features in a single fiber array. The second design primarily centers on octopus‐ and gecko‐inspired hybrid adhesive, which exhibits the benefits of both octopus‐ and gecko‐inspired microstructured adhesives for strong reversible adhesion on both wet and dry surfaces, such as skin. This fabrication approach could be used to produce many other 3D complex elastomeric structural adhesives for future real‐world applications.

## Graphical abstract

A 3D‐printing‐based fabrication approach is utilized for creating complex elastomeric microstructural adhesives directly. Two hybrid bioinspired structure designs are demonstrated, combining the strengths of gecko‐inspired microfiber adhesives with springtail‐ and octopus‐inspired microstructures to enable complex adhesives toward various real‐world applications.

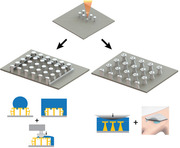

## 1 Introduction

Many synthetic advanced functional micro/nanomaterials are inspired by micro/nanostructured biological materials in nature as one of the promising approaches.^[^ ^1^, ^2^, ^3^ ^]^ These bioinspired functional micro/nanostructures have many different uses, such as dry adhesion,^[^ ^4^ ^]^ wet adhesion,^[^ ^5^ ^]^ liquid repellency,^[^ ^6^, ^7^, ^8^, ^9^ ^]^ and heat transfer.^[^ ^10^ ^]^ One of the widely studied bioinspired synthetic structures has been gecko‐foot‐hairs‐inspired dry fibrillar adhesives using majorly van der Waals forces to stick almost any smooth surface material.^[^ ^11^, ^12^, ^13^, ^14^, ^15^, ^16^, ^17^, ^18^ ^]^ These fibrillar repeatable and controllable adhesives have been well investigated in respect of contact mechanics,^[^ ^19^, ^20^ ^]^ adhesion and friction control,^[^ ^16^, ^21^, ^22^ ^]^ and wet and dry self‐cleaning.^[^ ^23^, ^24^, ^25^, ^26^ ^]^ Performance of these synthetic adhesives is even better than their biological counterparts source in some specific cases.^[^ ^27^, ^28^ ^]^ On the other hand, springtail‐inspired microstructures have been investigated for their liquid repellency.^[^ ^6^ ^]^ Springtail's skin can repel down to ≈25 mN m^–1^ surface tension liquids.^[^ ^29^ ^]^ Inspired by these microstructures on the springtail skin, some synthetic double re‐entrant microfibers were proposed to repel even fully wetting fluorinated liquids.^[^ ^6^ ^]^ Morphology of the gecko‐inspired T‐shaped fiber adhesives and springtail‐inspired double re‐entrant microfibers is similar with a flat fiber tip surface. However, springtail‐inspired structures have overhangs under their flat tip surfaces as different from the T‐shaped adhesives. Current double re‐entrant structures have been mostly made of rigid materials.^[^ ^6^, ^7^, ^8^, ^9^ ^]^ However, T‐shaped fibers are made of soft elastomers to attain conformal contact for high adhesion. A recent study merged these two concepts and showed both dry adhesion and super liquid repellency on the fiber top surface using elastomeric double re‐entrant microfibers with flat tips.^[^ ^30^ ^]^ Nevertheless, these fibers are sensitive to side wetting due to lack of their side‐surface liquid repellency.

Side‐surface liquid repellency can be possible by various methods. First, continuous sidewalls can be used,^[^ ^8^, ^31^, ^32^, ^33^ ^]^ which have the fundamental drawback of having their receding contact angle converge to zero degree at the walls during aspiration (dewetting).^[^ ^32^, ^33^, ^34^ ^]^ Here, the walls cannot repel the liquid after a certain point, because of high pinning forces due to a large contact area.^[^ ^8^, ^31^, ^32^, ^33^, ^34^ ^]^ Second, to overcome this problem, horizontal side double re‐entrant microfibers on the side surfaces can be used.^[^ ^7^ ^]^ As the main advantage of this method, the receding contact angle will be the same as the middle top‐surface part of the fiber array, thanks to the individual fiber placement on the boundary of the array, which decreases the contact area between the liquid and fibers dramatically. Such complex 3D structures cannot be fabricated by the typically used molding techniques;^[^ ^30^ ^]^ two‐photon polymerization type of 3D microprinting methods are required instead.^[^ ^7^ ^]^ Such horizontal side double re‐entrant microfibers were already 3D‐printed by rigid polymers so far for liquid repellency purposes only.^[^ ^7^ ^]^ Such rigid microstructures^[^ ^7^, ^8^, ^31^ ^]^ cannot be used as side liquid‐repellent dry fibrillar adhesives, which require soft elastomeric structures.^[^ ^35^ ^]^

On the other hand, several studies have developed elastomeric microstructured patches for attaching to skin or other tissues for medical and wearable device applications recently.^[^ ^36^, ^37^, ^38^, ^39^ ^]^ Achieving a conformal contact between the adhesive patch and biological surfaces is critical for high adhesion performance for tissue adhesives.^[^ ^40^, ^41^ ^]^ Gecko‐inspired fibrillar dry adhesives are one of the candidates for skin adhesion.^[^ ^41^, ^42^ ^]^ However, in wet, liquid‐immersed conditions, their adhesion is reduced notably because of significantly reduced van der Waals forces.^[^ ^43^ ^]^ On the other hand, octopus‐suction‐cup‐inspired microscale dome‐like protuberances adhere strongly in wet conditions on smooth surfaces.^[^ ^5^, ^44^ ^]^ But, they are not that effective in adhering to dry surfaces with compared to the gecko‐inspired adhesives. Therefore, combining gecko‐ and octopus‐inspired microstructured adhesives in the same elastomeric structures could be the most effective way to adhere strongly to wet and dry surfaces.

**Figure 1:**
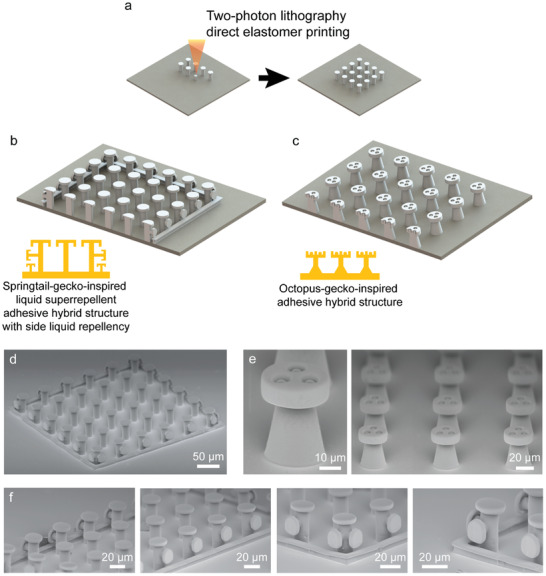
Direct‐3D‐printing‐based approach for fabricating elastomeric complex 3D bioinspired adhesives. a) Schematics of the fabrication process. Two‐photon‐polymerization‐based direct 3D printing of the structures using a custom elastomer resin. b,c) Inspiration sources and designs of two hybrid bioinspired adhesives. b) Springtail‐ and gecko‐inspired adhesive hybrid structures with side‐surface liquid repellency. c) Octopus‐ and gecko‐inspired adhesive hybrid structures with strong wet and dry adhesion. d–f) Scanning electron microscopy (SEM) images of the fabricated bioinspired adhesives. d) A full array of double re‐entrant structures with side‐surface liquid repellency. e) A single and an array of octopus–gecko‐inspired adhesive structures. f) Zoomed SEM images of the side and vertex structures of the springtail–gecko‐inspired adhesive structure array.

In this study, we used direct 3D printing of elastomeric microstructures using the two‐photon lithography technique (**Figure** **1**a) to enable two complex, 3D, and novel adhesive microstructure designs, which address the two open issues on side‐surface (in addition to top‐surface) liquid repellency (Figure 1b) and underwater adhesion of gecko‐inspired elastomeric microfiber adhesives (Figure 1c). First, combining both springtail‐ and gecko‐inspired microstructures into a hybrid structure (see Figure 1b,d,f) enabled side‐surface liquid‐repellent dry fibrillar adhesives. This microstructure design has three functionalities simultaneously: top‐surface liquid super‐repellency, side‐surface liquid‐repellency, and strong dry adhesion. Next, combining both octopus‐ and gecko‐inspired microstructures into a hybrid structure, as illustrated in Figure 1c,e, enabled high adhesion on both underwater and dry conditions on synthetic skin replicas toward future medical applications, merging the strength of each bioinspired structure design.

## 2 Results

**Figure 2:**
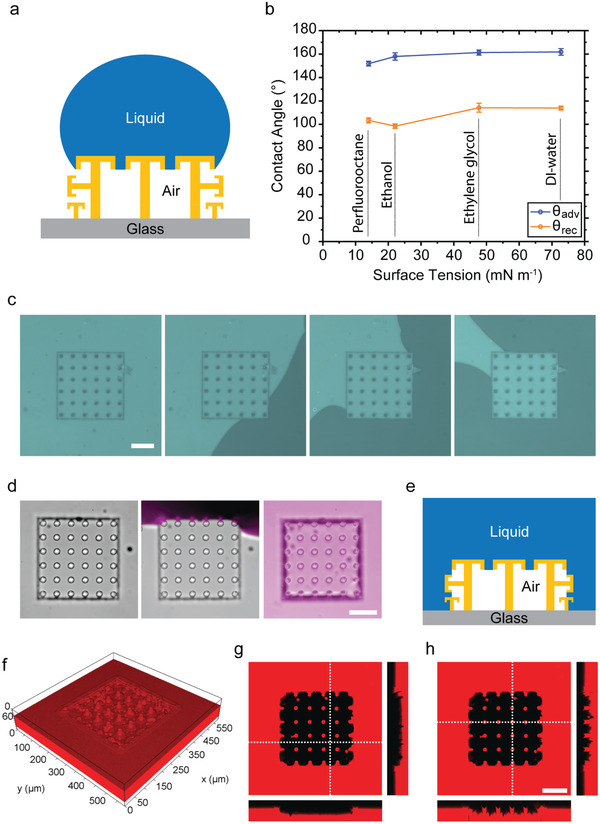
Top‐ and side‐surface liquid repellency of the springtail‐ and gecko‐inspired structures with a pitch distance of 60 µm. a) Schematic of a liquid droplet on top of the structures. b) Dynamic (advancing and receding) contact angles of different liquids including a fully wetting fluorinated liquid (perfluorooctane, γ ≈ 14.00 mN m^–1^). c) Only side‐surface liquid repellency of the springtail–gecko‐inspired adhesive structures. The green sections are the air and the black sections are the liquid regions. d–h) Top‐ and side‐surface liquid repellency performance of the full structure array in full immersion. d) The video snapshots while liquid approaches and covers all sides of the patch. e) Side‐view schematic of the patch in the full liquid immersion condition. f) 3D confocal optical microscopy image of these structures (upside‐down image to show the air cavity: the red section is the liquid part and the empty section is the air gap part) and g,h) 2D confocal microscopy cross‐section images of the array in full immersion. In confocal images, the red sections are the liquid and the black sections are the air parts. The repellent pillars are in contact with the red dye mixed inside the liquid. They reflect dye color; therefore, their stamp color is also red. Scale bars: 100 µm.

Top‐ and side‐surface liquid repellency of the springtail‐ and gecko‐inspired hybrid structure array was characterized using contact angle measurements. Diverse range of surface tension liquids was tested for advancing and receding contact angles. Surface tension of the liquids ranged between ≈14 and ≈72.80 mN m^–1^. One of the highly wetting liquids (perfluorooctane) could be repelled and remained in the Cassie state on the structures (**Figure** **2**a,b). Side‐surface liquid repellency of the array was also tested. For only side‐surface liquid repellency characterization, the top surface of the double‐reentrant fibers was covered by a glass slide. The liquid was applied into the pool with the constant rate of 1 mL min^–1^. As shown in Figure 2c, the fiber array repelled the deionized (DI) water from its side. In addition, top‐ and side‐surface liquid repellency of the array was tested under full liquid immersion (Figure 2d,e). For this experiment, we used a confocal microscope (Leica SP8, Wetzlar, Germany) to prove and visualize the liquid repellency in full‐immersion conditions. As shown in the confocal microscopy images in Figure 2f–h, the springtail‐ and gecko‐inspired patch was able to repel liquid from its top surface and all side surfaces when it was fully immersed with DI water. During full immersion experiments, the rate of the applied liquid was 1 mL min^–1^.

**Figure 3:**
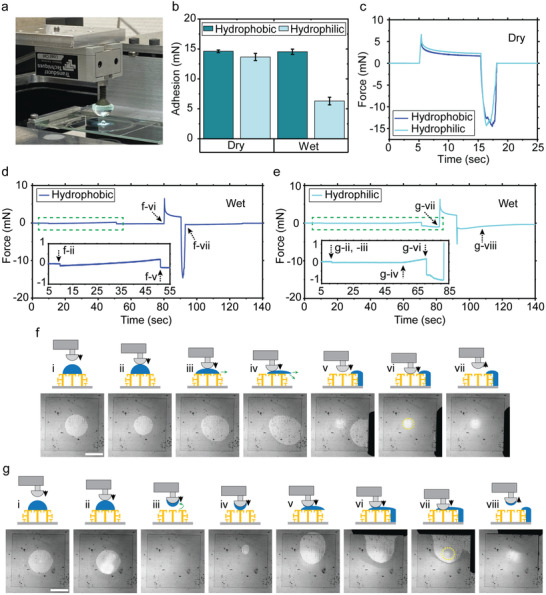
Adhesion characterization results of the springtail–gecko‐inspired structures with side‐surface liquid repellency and pitch distance of 60 µm. a) A picture of the custom adhesion set‐up with a droplet on top of the sample. b) Dry and wet adhesion results using both hydrophobic and hydrophilic hemisphere glass probes. c) Representative dry adhesion force graphs with respect to time for the hydrophobic and hydrophilic probes. d,e) Representative wet adhesion force graphs with respect to time for the hydrophobic probe (d) and the hydrophilic probe (e). f,g) Schematic and experimental video snapshots during wet adhesion testing, where the side liquid repellency is observed when using the hydrophobic probe (f) and the hydrophilic probe (g). All the error bars in graphs represent standard deviations for samples (*N* = 5). Scale bars: 1 mm.

The hybrid structure array's adhesion performance was characterized with a uniaxial adhesion setup (**Figure** **3**a). During these measurements, a smooth hemispherical glass (diameter 10 mm) was used to contact with the structure top surfaces. Approaching and retracting speeds were 25 µm s^–1^ during these tests. Initially, the saturation value of the preload was found for these structures (Figure S4, Supporting Information). The rest of the adhesion experiments were performed using the same saturation preload value (6 mN). For adhesion tests, hydrophobic and hydrophilic glass probes were used to investigate the effect of contact surface's wettability on adhesion in both dry and wet conditions. Initially, dry adhesion tests were performed for hydrophilic and hydrophobic probes. Both different wettability behavior probes resulted in almost the same adhesion performances in dry conditions (Figure 3b,c). For wet adhesion characterization (Figure 3d,e), we applied a 5 µL DI‐water droplet on the top surface of the patch. Additionally, during hydrophobic and hydrophilic probe adhesion tests, droplet always stayed in the Cassie regime on the patch. The results showed that the hydrophobic probe was able to push the liquid to the side, before contacting the fiber tip surfaces (Figure 3f). Thus, a dry contact was possible with the double re‐entrant fiber tips after pushing droplet out of the patch. As a result, in wet conditions, the hydrophobic probe performed the similar adhesion performance with dry conditions (Figure 3b,d).^[^ ^30^ ^]^ The side liquid‐repellent structures pushed away from the droplets and the liquid could not penetrate inside of the patch when they passed out of the patch area (Figure 3f).^[^ ^7^ ^]^

For wet adhesion experiments with the hydrophilic glass probe, while the probe was approaching the fibers, the complete droplet moved to the glass probe just after initial contact. Afterward, no liquid remained on the patch top surface (Figure 3e,g) due to the high wettability behavior of the hydrophilic probe and liquid super‐repellency of the patch. The initial contact between the fiber tips and the probe occurred while the hydrophilic probe was carrying the droplet. At the end of the approaching state, the droplets got wider between the probe and fibers. During preloading, the droplets could not be pushed away completely due to high wettability of the hydrophilic probe. The droplets remained between the fibers and hydrophilic probe. Consequently, the hydrophilic probe resulted in relatively lower adhesion values compared to the dry case, due to the liquid layer between the two interfaces (Figure 3b,e,g). During these experiments, similar to the hydrophobic probe adhesion tests, pushed‐out liquid droplets did not penetrate into the patch because of the side liquid‐repellent structures (Figure 3g). In these tests, we observed the advantage of having all three features (top‐surface liquid repellency, side‐surface liquid repellency, and strong adhesion) at the same time.

To compare these adhesion performance results with the literature, the researchers reported that biological gecko foot hairs have around 10 kPa normal and 100 kPa shear adhesive strength on smooth glass surfaces.^[^ ^4^, ^45^ ^]^ As single‐material‐based synthetic high‐performance gecko‐inspired adhesives, different groups^[^ ^13^, ^14^ ^]^ reported elastomeric gecko‐inspired mushroom structures, which had 100–180 kPa adhesion strength on a dry smooth glass substrate. Recently, another study showed that liquid‐repellent adhesive structures had ≈100 kPa adhesive strength.^[^ ^30^ ^]^ In this study, we showed that our springtail‐ and gecko‐inspired adhesive structures had contact area with ≈200 µm contact radius after contacting to a smooth glass hemisphere. The full contact area was 0.124 (± 0.015) mm^2^. The average dry adhesion force was measured as 14.3 (± 0.5) mN. Thus, we achieved the dry adhesive strength of 115 kPa, similar to high‐performance gecko‐inspired microfiber adhesives in the literature.

**Figure 4:**
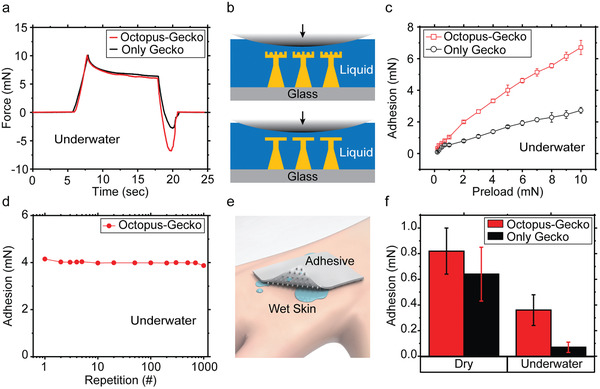
Adhesion characterization results of the hybrid octopus–gecko‐inspired structure patch with pitch distance of 80 µm. a) Representative adhesion‐force–time graphs for only‐gecko‐inspired and hybrid octopus–gecko‐inspired structures in immersed conditions with DI‐water. The applied preload was 10 mN in ambient conditions and the approaching and retraction speeds were 5 µm s^–1^ and the relaxation time was set to 10 s. b) Schematic illustrations of the only‐gecko‐inspired structure (bottom) and the hybrid octopus–gecko‐inspired structure (top) for adhesion under water. c) Adhesion values for different preloads (0.2–10 mN) in underwater condition. d) Repeatability adhesion tests of the hybrid octopus–gecko‐inspired structure more than 1000 cycles. The applied preload was 5 mN and the time interval between measurements was 5 s. e) Schematic of the hybrid structure patch adhering to the wet and rough biological skin. f) Adhesion results for only‐gecko‐inspired patch and hybrid structured patch in dry and underwater conditions on a synthetic skin replica with the preload of 5 mN. For measurements, the skin replica was placed into a container without or with DI‐ water on the stage, and then the flat end screw attached with the hybrid‐structured patch was moved to contact with the skin replica. All the error bars in the graphs represent standard deviations for the samples (*N* = 5).

Next, wet and dry adhesion performance of the octopus‐ and gecko‐inspired hybrid microstructures was characterized (**Figure** **4**). It is well known that the octopus patterns are effective to achieve strong wet adhesion.^[^ ^5^, ^44^, ^46^, ^47^ ^]^ Specifically, the dome‐like protuberance structure of the octopus suckers enhance wet adhesion due to their structural property.^[^ ^5^, ^44^, ^48^ ^]^ As shown in Figure 4a, gecko‐inspired microfiber adhesives showed low underwater adhesion performance due to the significant reduction of the van der Waals forces in immersed conditions.^[^ ^39^ ^]^ This is the result of the interfacial liquid layer between the glass probe and the microfiber tip surfaces. On the other hand, inserting the octopus patterns on the microfiber tip surfaces increased the wet adhesion with an applied preload by generating a cohesive force among the liquid molecules without an insignificant energy consumption owing to the internal dome‐like protuberance structure that makes the residual liquid in the chamber to pull up to both sides due to capillary force induced by deformation with vertical preload (Figure 4a,b).^[^ ^5^ ^]^ Thus, these hybrid structures exhibited higher underwater adhesion than the gecko‐inspired adhesives (Figure 4c). By increasing the preload from 0.2 to 10 mN, the adhesion performance increased around three times under water. Thus, the hybrid design improved the underwater adhesion while maintaining the dry adhesion performance of the gecko‐inspired microfibers. In dry conditions, the gecko‐inspired structure had stronger adhesive force than the octopus‐ and gecko‐inspired hybrid structure because of larger contact area (Figure S6, Supporting Information). In addition, the operational reliability of adhesion performance in underwater condition was confirmed in Figure 4d. The measurements were conducted by repeatedly applying the vertical preload (5 mN) on the hybrid structures with over 1000 attachment‐detachment cycles, where the approaching and retraction speeds were 5 µm s^–1^ and the relaxation time was 10 s, and the time interval between measurements was 5 s. The results show that the adhesion performance was highly robust for octopus‐ and gecko‐inspired hybrid structures in underwater conditions. Likewise, in dry environments with the same measurement conditions (preload of 5 mN), the hybrid structure also showed a reproducible adhesion performance under 1000 loading‐to‐unloading cycles (Figure S7, Supporting Information) because the printed elastomer material is highly robust to preload owing to its mechanical flexibility.

Adhesion performance of the octopus–gecko‐inspired patch on a synthetic soft skin replica (with a similar mechanical and structural property of a biological skin) was characterized in both dry and underwater conditions (Figure 4e,f). In the dry condition, the octopus–gecko‐inspired patch presented higher adhesion than the gecko‐inspired patch on the synthetic soft skin. This means that the adhesion improved due to the suction effect. Octopus patterns had a high suction force on the rough skin surface. On the other hand, gecko‐inspired patches were supposed to have lower fiber tip‐surface contact area on rough surfaces. This leads to lower adhesion compared to the octopus–gecko‐inspired hybrid adhesives in both dry and wet conditions on the skin replica. More importantly, the gecko‐inspired patch almost lost its adhesion underwater. In comparison, the octopus–gecko‐inspired hybrid structure showed around three times higher adhesion and high repeatability under water. Therefore, the octopus–gecko‐inspired structures overcame the shortcomings and showed high adhesion on both wet and dry skin replica.

## 3 Conclusion

Our approach of the direct 3D printing of the elastomeric 3D microstructures allowed us to fabricate hybrid, bioinspired, multifunctional, and complex adhesive structures. The demonstrated two‐hybrid adhesive designs improved the performance of the current structural adhesives in different real‐world conditions. This approach enables to integrate many other different bioinspired or human‐made, 3D and complex structural adhesives almost without considering any fabrication constraint. The first demonstrated array design showed top‐surface liquid super‐repellency, side‐surface liquid repellency, and strong adhesion on the same patch for the first time. The second design enabled strong adhesion in both underwater and dry conditions. Low Young's modulus, high elongation, and high surface energy of the custom aliphatic urethane‐acrylate‐based elastomer were essential for these complex structural adhesives to have high performance. In terms of the fabrication speed and throughput, this approach may not be comparable with the molding techniques. However, the proposed approach is needed to fabricate complex 3D elastomeric structural adhesives, which cannot be molded reliably. The speed and throughput of the fabrication process will be enhanced proportionally by the advancements in the commercial two‐photon lithography systems. Already, there are some two‐photon‐lithography systems in the market for high‐throughput production for industrial use. Furthermore, there are many investigations to increase the throughput and speed of two‐photon lithography in industry. The proposed approach can be also used in developing other future complex structural elastomer adhesives and other microstructured 3D materials. These complex adhesive structures can be used in robotics, biomedical device, part and tissue handling, fastener, and pick‐and‐place applications in dry and wet conditions. For these potential applications, the required patch areas can vary from hundreds of micrometers to several centimeters square, where large‐area samples would take more fabrication time up to a day. Therefore, tissue handling, electronic device component handling, biomedical device, and robotic applications requiring only small‐area adhesive patches would be a better fit for shorter fabrication times of several hours.

## 4 Experimental Section

### Custom‐made Elastomeric Resin Material

A custom‐made photocurable resin was used as an elastomeric material to be used in two‐photon‐lithography‐based 3D printing of the hybrid structure designs. The material is made of an oligomer, a monomer, and a photoinitiator. BASF Laromer UA 9072, which is a urethane‐modified acrylic resin, was used as the oligomer with 92 wt%. As the monomer, bisphenol A ethoxylate dimethacrylate 15 (BPA(EO)15DMA) was included with 5 wt%. Diphenyl (2,4,6‐trimethylbenzoyl) phosphine oxide (TPO) with 3 wt% was added as the photoinitiator (**Table** **1**). All these ingredients were mixed for ≈16 h with 50 rpm using magnetic stirrer (RCT basic, IKA, Germany) until the resin was homogenous.

**Table 1:** The custom aliphatic urethane‐acrylate‐based elastomer resin material composition that was used for two‐photon‐polymerization‐based elastomeric microstructure 3D printing

Materials	Chemistry	Trade name	Concentration [wt%]
Oligomer	Aliphatic urethane acrylate	BASF Laromer UA 9072	92
Monomer	Bisphenol‐A‐ethoxylate15‐dimethacrylate	–	5
Photoinitiator	Diphenyl‐(2,4,6‐trimethylbenzoyl)‐phosphine oxide	TPO	3

The Young's modulus and elongation at break of the cured custom resin material were measured as 17.1 ± 2.2 MPa and 126.3 ± 19.4%, respectively using a universal tensile testing machine (model: 5942, INSTRON, Norwood, MA, USA) according to the ISO‐527‐2‐type‐5b standard.^[^ ^49^ ^]^ The surface energy of the cured resin was calculated as 40.4 mN m^−1^, according to the Fowkes model.

### Fabrication Process of the Microstructures

For fabricating the elastomeric bioinspired hybrid adhesive microstructures, direct elastomer 3D printing using the two‐photon‐polymerization technique was implemented. After the printing of the desired structures using the custom‐made resin, they were subjected to post‐processing. The samples were immersed in a beaker containing propylene glycol methyl ether acetate (PGMEA) for 2 h to dissolve the uncured parts of the resin. Next, the samples were placed for 5 min in another beaker containing fresh PGMEA to ensure the uncured parts of the resin was dissolved completely. Then, the samples were placed in a beaker containing isopropanol alcohol (IPA) for 3 min to terminate the dissolving process. As the next step, the samples were transferred into fresh IPA and post‐UV curing was done for 3 min with an external UV‐curing system (Omnicure Series 2000, Excelitas Tech. Corp.). Finally, the samples were dried using a critical point dryer (Leica EM CPD300, Wetzlar, Germany).

### Springtail‐ and Gecko‐Inspired Hybrid Microstructure Geometries

The tip diameter of the double re‐entrant structure's top part was 30 µm, the stamp diameter was set to 18 µm, the tip thickness was set to 3 µm, the overhang thickness was 2 µm, and the overhang height was 3 µm. For outer boundary structures of the array (double re‐entrant structures with side double re‐entrant branches), the top part was identical with the tip of the middle structure (there is no side branched double re‐entrant structures for the middle fibers). Additionally, the patch array had outer boundary double re‐entrant fibers with side double re‐entrant branches to repel liquids from side. For side double re‐entrant branches, the tip diameter was set to 22 µm, the stamp diameter was 10 µm, the tip thickness was 3 µm, the overhang thickness was 2 µm, and the overhang height was 3 µm. For all fibers, the structure height was 50 µm (Figure S3, Supporting Information). The pitch distance was 60 µm among fibers. Furthermore, boundary structures also had a continuous wall below their side double reentrant branches. These small continuous walls were necessary to keep liquids outside of the patch. The absence of small continuous walls below side double‐reentrant branches cause liquid to proceed on the glass and penetrate inside of the patch area.^[^ ^7^ ^]^

### Octopus‐ and Gecko‐Inspired Hybrid Microstructure Geometries

Tip diameter of these fibrillar structures was 36 µm, the tip thickness was 10 µm, the stamp neck diameter was 20 µm, the base diameter was 30 µm, and the height of the structure was 47 µm for octopus‐ and gecko‐inspired fiber structures and T‐shape fibrils. The pitch distance among fibers was 80 µm for both structure geometries. The protuberance diameter of octopus‐ and gecko‐inspired fiber structure geometry was 7 µm and it was shifted 1 µm downward for attaching protuberance inside of the suction cup. The suction cup diameter was 10 µm with 7 µm height. For each octopus‐ and gecko‐ inspired fiber structures, three octopus inspired suction cups were placed on top of the tip with 8 µm spacing in each (Figure S3, Supporting Information).

### Direct 3D Printing of the Elastomeric Hybrid Fibrillar Microstructures

For each individual structure, computer‐aided design was realized by Solidworks. Then the stereolithography file (.stl) was generated. The generated file (.stl) was loaded into the Nanoscribe software (Photonic Professional GT2, Nanoscribe GmbH, Germany). Here, the two‐photon lithography system was used in oil mode. In this mode, the resin material was placed on top of the glass and the oil was placed between the glass and the objective. As the objective lens, 63x, 1.4 NA objective was used for achieving high resolution. Galvo scan mode was preferred to print the structures layer by layer. For the resin, the ideal process parameters were found by optimizing the process parameters, especially the scan speed and laser power. The optimum parameters were 75% for the laser power and 6 mm s^–1^ for the scan speed. The fabrication duration of a single structure is between 30 s and 1 min depending on the complexity of the 3D fibril shape.

### Wettability Characterization of the Springtail–Gecko‐Inspired Structures

To determine the surface wettability of the springtail–gecko‐inspired structures, a commercial contact angle measurement device (Drop Shape Analyzer DSA100, Krüss GmbH, Hamburg, Germany) was used. As the characterization method, the sessile drop was chosen. For each liquid, advancing and receding contact angles were measured at least 10 times. For each characterization, applied droplet volume varied between ≈2 and ≈5 µL. As the liquid dosing and aspiration speed, ≈0.2 µL s^–1^ was used. All these measurements were performed in room conditions with 23 °C temperature and 30% humidity.

### Full Immersion and Only Side‐Surface Liquid Repellency Characterization of the Springtail–Gecko‐Inspired Structures

For full immersion and only side liquid repellency characterizations of springtail–gecko‐inspired structures, the sample was placed on a glass microscope slide. The microscope slide boundary was covered by a poly(methyl methacrylate) (PMMA) wall. One side of the polymer tube was attached inside of the pool and the other end was attached to a 20 mL plastic syringe. A plastic syringe was placed on a programmable syringe pump (Legato 210p, KD Scientific, USA) to apply the liquid in a controlled and robust rate. For full immersion characterizations, the liquid was applied with a constant rate of 1 mL min^–1^ until to reach 5 mm height of liquid inside the pool. For only side‐surface wetting characterizations, a flat untreated glass was placed on the patch before applying the liquid. Then, the liquid was applied with the rate of 1 mL min^–1^ until all side of the patch was covered by the liquid. The applied liquid height did not pass top part of the glass during these experiments. All these measurements were carried out in room conditions with 23°C temperature and 30% humidity.

### Confocal Optical Microscopy Imaging

For the visualization of immersion, the samples were placed inside a PMMA wall and filled with water using a syringe pump (Legato 210p, KD Scientific, USA) at a rate of 1 mL min^–1^. For fluorescence imaging, Rhodamine B fluorescent dye (0.001 mg mL^−1^ in dH_2_O) was used. Samples were imaged and recorded during the immersion experiment to show liquid‐structure interactions with a Leica DMi8 fluorescence inverted microscope (Wetzlar, Germany). Images, where liquid‐structure interactions take place, were pseudo‐colored using Adobe Photoshop software. A Leica SP8 single‐point scanning confocal microscope (Wetzlar, Germany) equipped with a 20×/0.4 objective was used to obtain confocal images of structures and liquid–vapor interfaces immediately after immersion. A 3D reconstruction of the *z*‐stack planes was made using LAS X software. All these measurements were carried out in room conditions with 23 °C temperature and 30% humidity.

### Adhesion Characterization Setup

A custom‐made adhesion characterization setup was used for the dry and wet adhesion measurements. To visualize and record the contact, the video camera (Grasshopper3, Point Grey Research Inc.) was connected to an inverted optical microscope (Axio Observer A1, Zeiss). A computer‐controlled high precision piezo stage (LPS‐65 2”, Physik Instrumente GmbH & Co. KG) was mounted on the microscope for *z*‐direction. The resolution of the motion stage was 5 nm. For alignment in the *x* and *y* directions, the manual stage (NFP‐2462CC, Positionierungstechnik Dr Meierling) was used. To find adjustments for tilting was done by two goniometers (M‐GON65‐U, Newport, Irvine, CA, USA). A sensitive load cell (GSO‐25, Transducer Techniques, Temecula, CA, USA) was mounted on the piezo motion *z*‐stage to obtain force data. A signal conditioner (TMO‐2, Transducer Techniques, Temecula, CA, USA) and data acquisition board (USB‐6001, National Instruments, Austin, TX, USA) were connected to the load cell for computer connection. The data acquisition and motion control of the piezo stage were managed by a custom‐made LabVIEW (National Instruments, Austin, TX, USA) program. Preloads, velocities, contact times, and the displacements in the *z* direction were controlled using the program.

### Adhesion Testing

The load cell of the adhesion setup was connected to a flat end screw. A flat end screw glued on the flat side of the glass hemisphere probe (10 mm diameter, ACL108U, Thorlabs). The glass hemisphere probe was used as a contact surface during the measurements. The microfiber adhesive patches were placed on a microscope slide.

For the springtail–gecko‐inspired adhesives, the approaching speed was 25 µm s^–1^. After reaching the desired preload, the relaxation time was 10 s during all measurements. Then, until the glass probe fully detached, the probe was retracted at 25 µm s^–1^. For wet adhesion characterizations, the amount of the DI‐water droplet was 5 µL. The lower amount of the liquid was not possible to apply due to the super‐liquid‐repellent property of these structures.

For all octopus–gecko‐inspired and gecko‐inspired structure adhesion experiments, the approaching and retraction speeds were 5 µm s^–1^ and the relaxation time was set to 10 s. A representative force–time measurement (Figure 4a), dry and underwater adhesion with different preloads measurements (Figure 4c and Figure S6, Supporting Information), adhesion repeatability characterizations (Figure 4d) were conducted by using the glass hemisphere probe for different preload adhesion measurements. For the hemisphere glass probe adhesions, the glass probe approached to and retracted from the patch sample while force values were measured from the load cell. For all skin replica experiments, the hybrid or gecko‐inspired adhesive patch was glued to the load cell with a tungsten connector. For dry skin adhesion measurements, the skin replica was placed on the stage without any liquid in the environment. For underwater skin adhesion measurements, the skin replica was placed on the stage into a DI‐water filled container. In all skin adhesion measurements, while the force values were measuring from the load cell, the hybrid or gecko‐inspired adhesive patch approached to and retracted from the skin replica.

To minimize the viscoelastic effects, approach and retraction velocities were set to low values for all adhesion experiments. After each set of measurements, the probe was cleaned with particle‐free tissue and isopropyl alcohol. For each data point, experiments were repeated at least five times. All these measurements were carried out in room conditions with 23 °C temperature and 30% humidity.

## Conflict of Interest

The authors declare no conflict of interest.

## Supporting information

Supporting Information

Supplemental Movie 1

Supplemental Movie 2

Supplemental Movie 3

Supplemental Movie 4

Supplemental Movie 5

Supplemental Movie 6

Supplemental Movie 7

## Data Availability

The data that support the findings of this study are available from the corresponding author upon reasonable request.
