# Supplementary material for: 3D Printing of Elastomeric Bioinspired Complex Adhesive Microstructures
Source: Adv Mater. 2021 Aug 15;33(40):2103826. doi: 10.1002/adma.202103826 (PMC11468481; doi:10.1002/adma.202103826)
Supplement: Supplementary file 1 — Supporting Information [file ADMA-33-2103826-s003.pdf]

© 2021 Wiley-VCH GmbH

ADVANCED MATERIALS

Supporting Information

for *Adv. Mater.,* DOI: 10.1002/adma.202103826

3D Printing of Elastomeric Bioinspired Complex Adhesive Microstructures

*Cem Balda Dayan, Sungwoo Chun, Nagaraj Krishna-Subbaiah, Dirk-Michael Drotlef, Mukrime Birgul Akolpoglu, and Metin Sitti**

Supporting Information

3D Printing of Elastomeric Bioinspired Complex Adhesive Microstructures

Cem Balda Dayan, Sungwoo Chun, Nagaraj Krishna-Subbaiah, Dirk-Michael Drotlef,
Mukrime Birgul Akolpoglu, Metin Sitti

Liquid repellency modeling:

If a liquid is repelled by structural array (a droplet in the Cassie state), the apparent contact angle (θ^*) is defined by the Cassie-Baxter model[1] as:

$$\cos\theta^* = f_s \cos\theta_Y - f_g \quad (1)$$

where θ_Y is the intrinsic contact angle (i.e., the contact angle between a smooth solid surface and a liquid), f_s is the solid fraction that is the solid-liquid contact area (solid-liquid interface) divided by the total projected area, and f_g is the gas fraction that is the air-liquid area (air-liquid interface) divided by the total projected area. In the ideal Cassie state, the solid-liquid and air-liquid interfaces are completely flat ($f_s + f_g = 1$) and this simplifies the Equation 1 as:[2]

$$\cos\theta^* = f_s (1+\cos\theta_Y) - 1 \quad (2)$$

which explains the relation between θ^*, f_s, and θ_Y. Increase in the apparent contact angle means improving the liquid repellency. If the solid fraction decreases or the intrinsic contact angle increases, the apparent contact angle rises. If the solid fraction decreases, the effect of the intrinsic contact angle on the apparent contact angle also reduces. The impact of the material's internal wettability characteristic (related to θ_Y) on liquid repellency (related to θ^*) declines if the solid fraction decreases as shown in **Figure S9**. This means that if the solid fraction has a lower value, regardless of the material, the structured surfaces can repel extremely low surface tension liquids.[2] In terms of the structure dimensions, the solid fraction can be reduced by decreasing the tip diameter, the tip thickness, the overhang height, and the overhang thickness, in contrast to increasing the pitch distance.

Not only the solid fraction, but also the shape of the structure is important for liquid repellency and this is determined by the critical angle (θ_c). The structured surfaces can repel liquids only if the critical angle is larger than 180^0. The critical angle is the summation of the edge angle (θ_0) of the structure and the intrinsic contact angle (θ_Y) where[3]

$$\theta_c = \theta_0 + \theta_Y . \quad (3)$$

The edge angle varies if the shape of the structure changes. As an example, the edge angle of the pillar structure is 90^0. This means that the intrinsic contact angle larger than 90^0 liquids can be repelled by the pillar structures. For that reason, fully wetting liquids ($\theta_Y \sim 0^0$) cannot be repelled by the pillar structures. For the T-shaped structures, the edge angle is 180^0, which means that larger than 0^0 intrinsic contact angle liquids can be repelled in theory. This value is exactly on the critical limit for fully wetting liquids. Due to just being on the limit, any imperfection can disturb the liquid repellency property in practice. However, for double re-

entrant structures, the edge angle is 270^0. This means that even fully wetting (the intrinsic contact angle 0^0) liquids can be repelled because their edge angle is much more than the critical limit (180^0). The edge angle measurements of different structures are reported in **Figure S10**.[3]

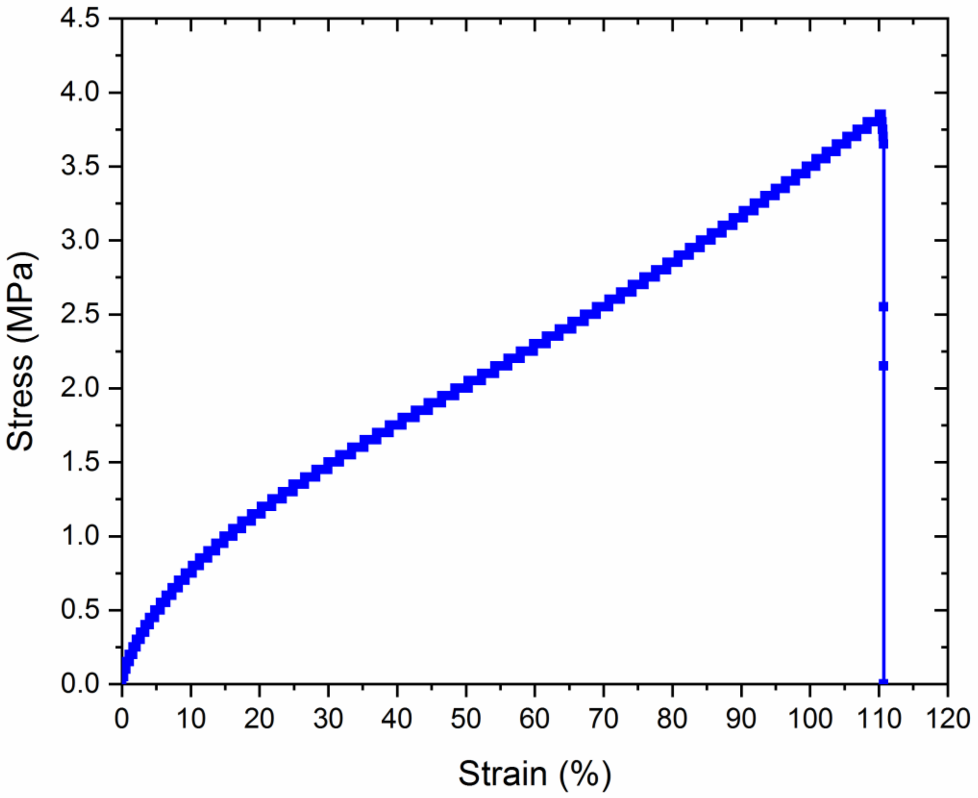

Figure S1. Stress-strain test result of the custom resin material (see Table 1 for the composition details).

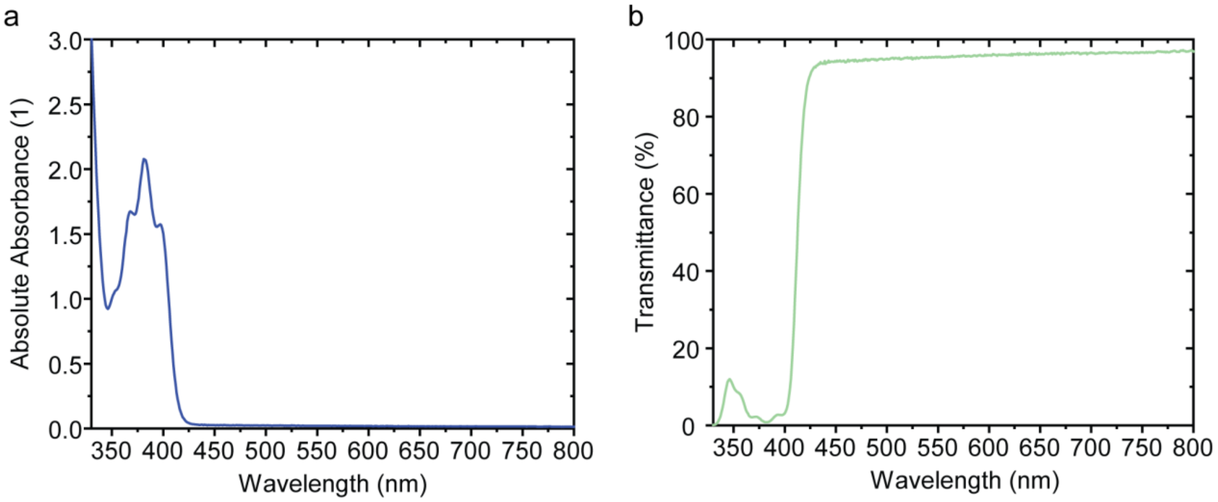

Figure S2. a) Absorbance vs. wavelength and b) transmittance vs. wavelength graphs for the custom resin.

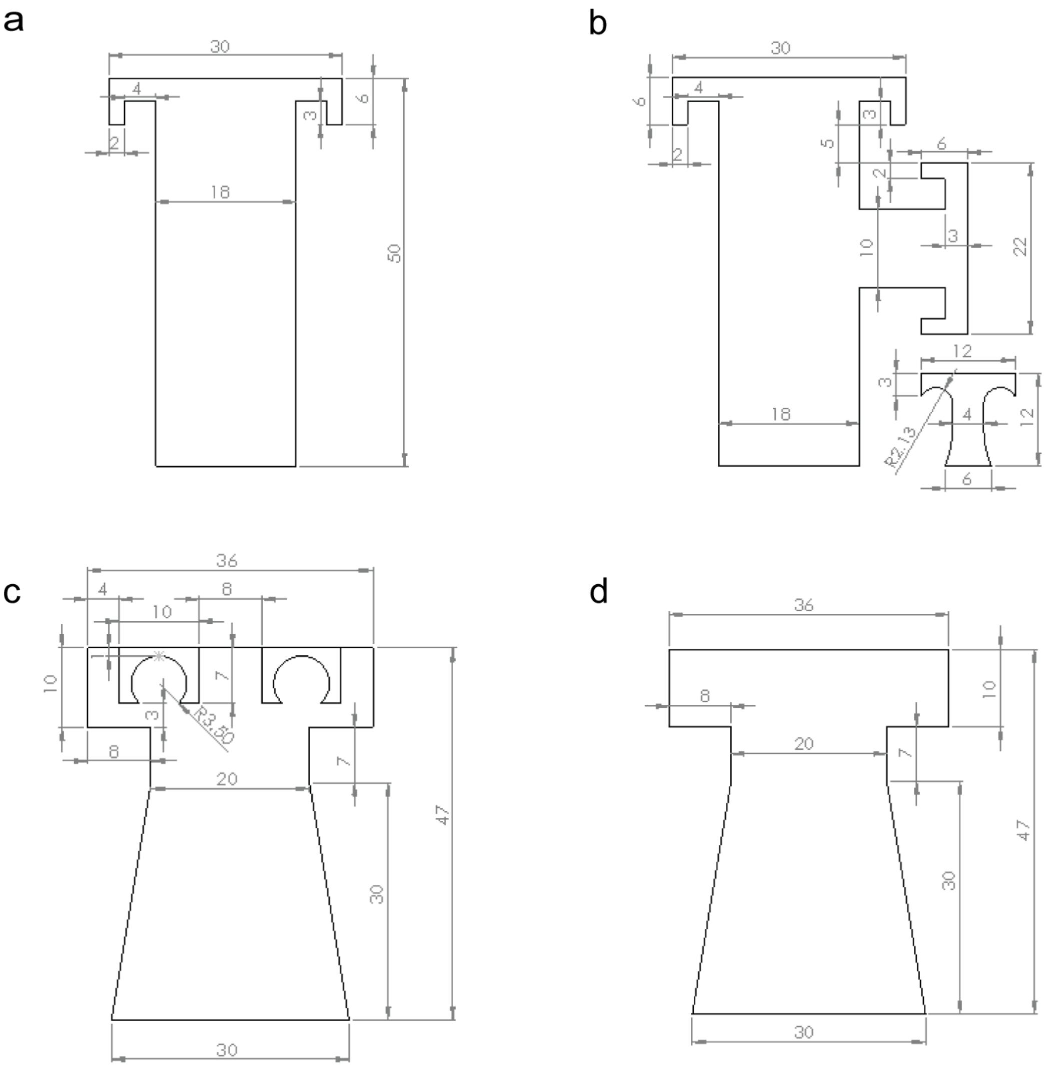

Figure S3. Dimensions of all demonstrated adhesive structures in this study: a) middle and b) side structures of the springtail-gecko-inspired array, c) the octopus-gecko-inspired and d) the gecko-inspired fibril adhesive structures.

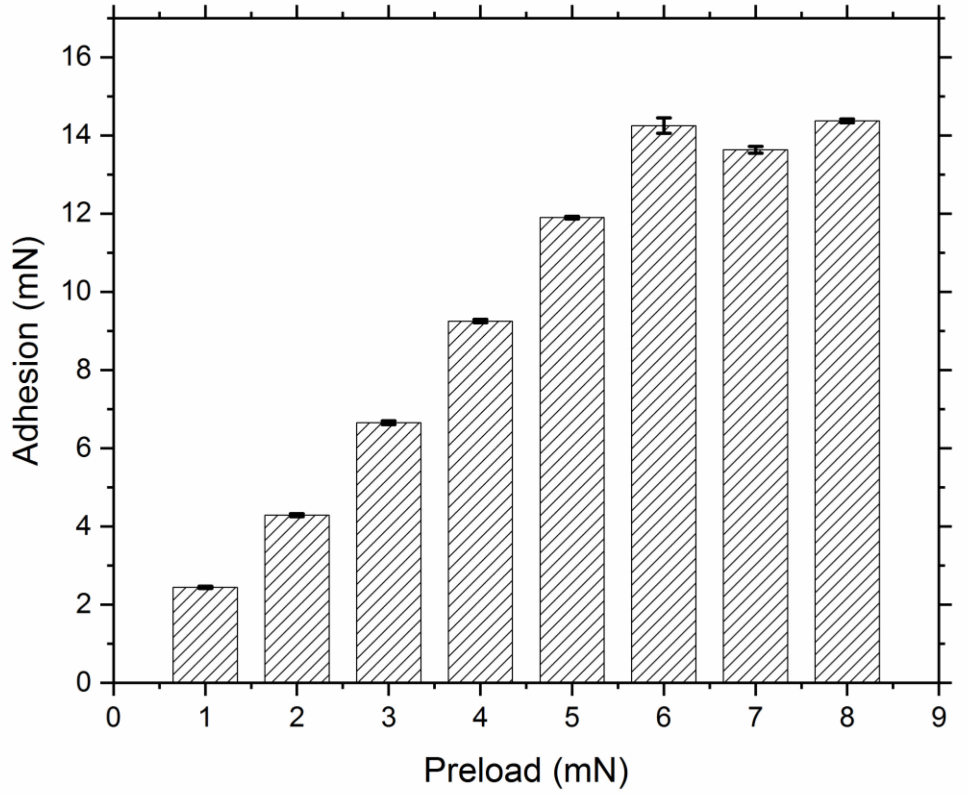

Figure S4. Saturation preload test for the double re-entrant structure adhesives on a hemispherical smooth glass indenter (approaching and retraction speed: 25 μm s^{-1}, relaxation time after preloading: 10 s). The observed saturation preload is 6 mN.

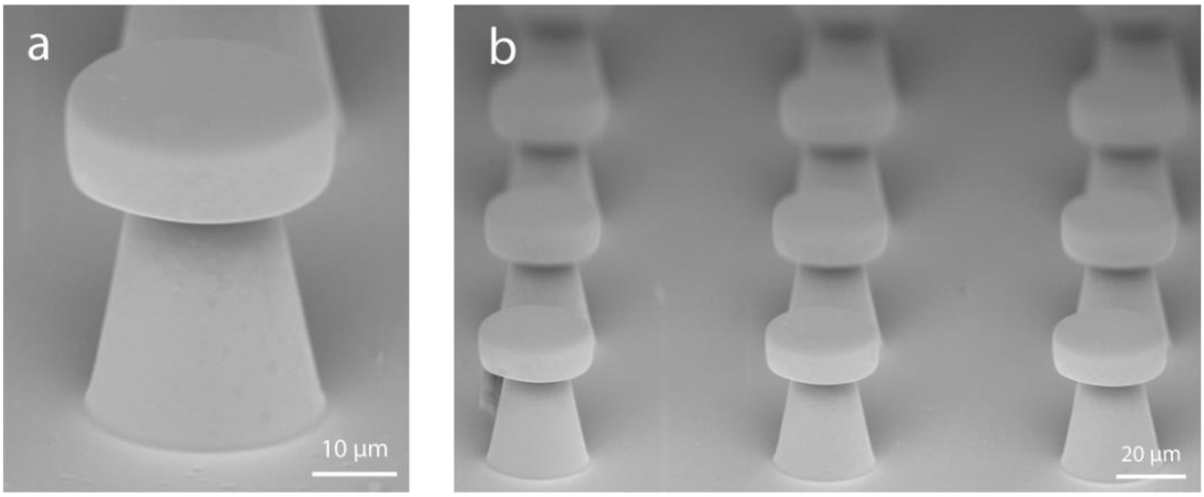

Figure S5. SEM images of the gecko-inspired fiber adhesives: a) the single fiber structure and b) the fiber array.

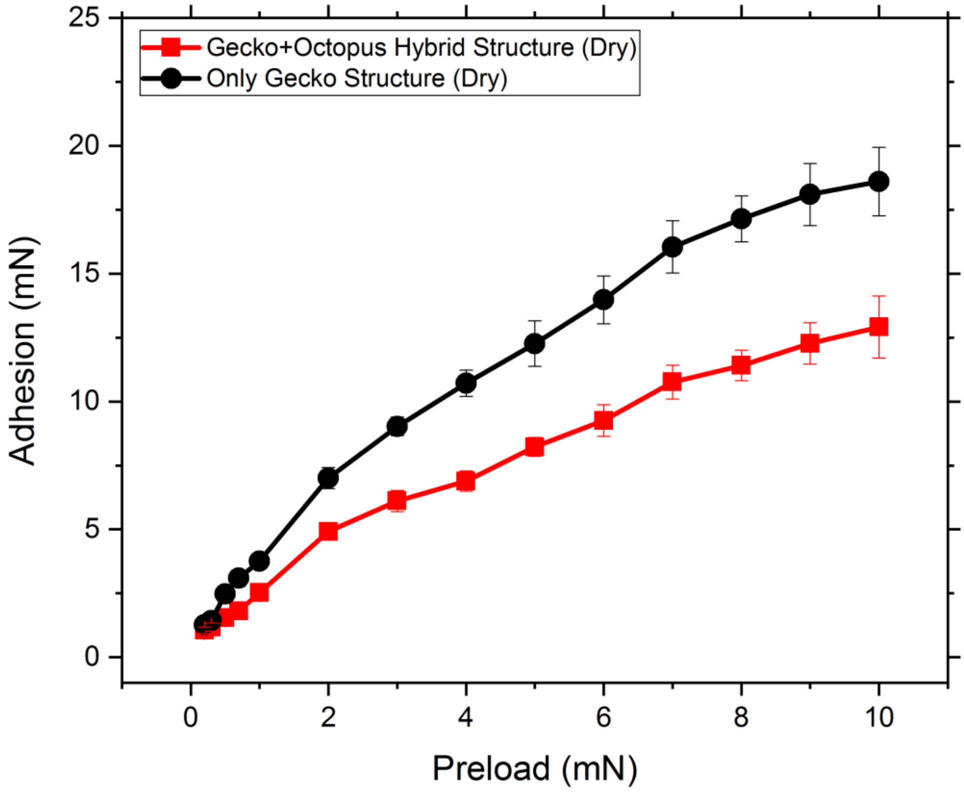

Figure S6. Dry adhesion test results of the octopus-gecko-inspired and the gecko-inspired structures under different preloads using a hemispherical smooth glass indenter.

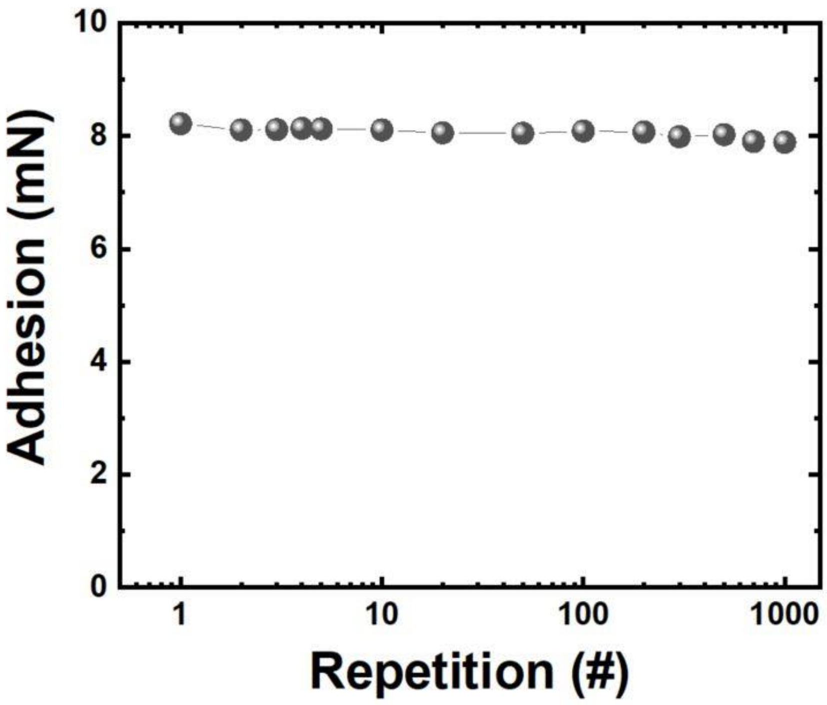

Figure S7. Reproducible adhesion performance of the hybrid octopus-gecko-inspired structures in dry conditions in more than 1,000 loading-to-unloading cycles with the preload of 5 mN, motion speed of 5 μm s^{-1}, and relaxation time of 10 s after preloading. The waiting time between each test cycle was 5 s.

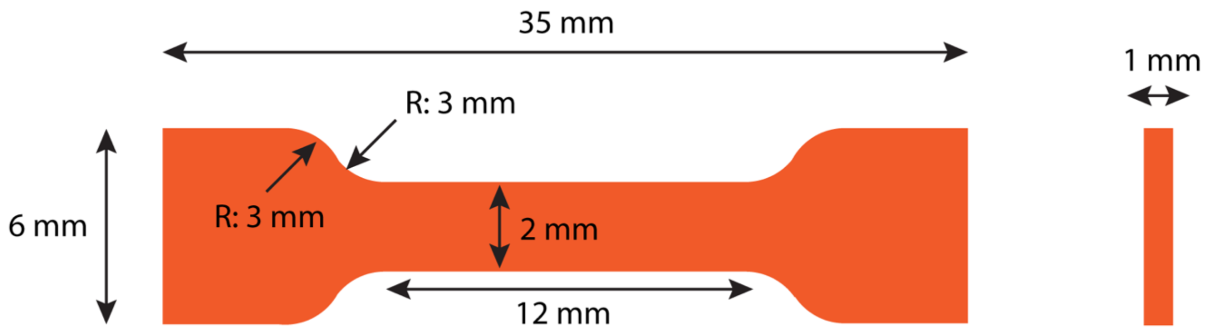

Figure S8. The specimen shape and dimensions for custom resin's mechanical characterization according to the ISO-527-2 standards.

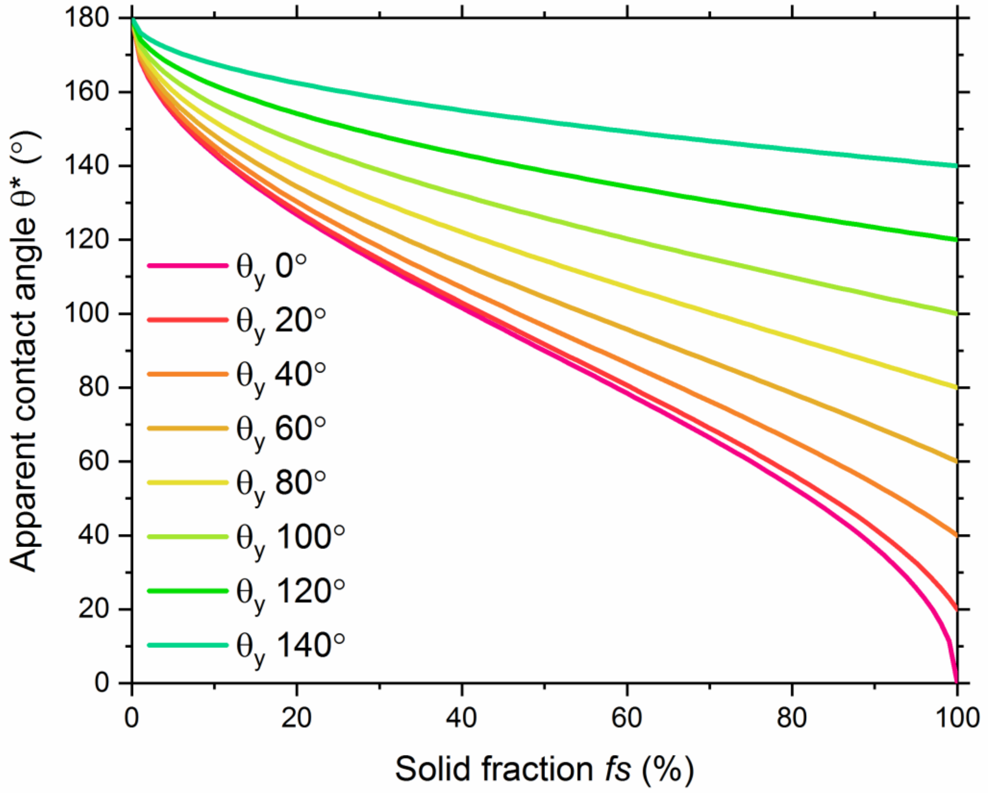

Figure S9. The effect of the solid fraction (f_s) on the apparent contact angle (θ^*) for variety of intrinsic contact angles (θ_Y). The solid fraction is determined by the structural dimensions (such as the tip diameter, the tip thickness, the overhang height, the overhang thickness, and the pitch distance).

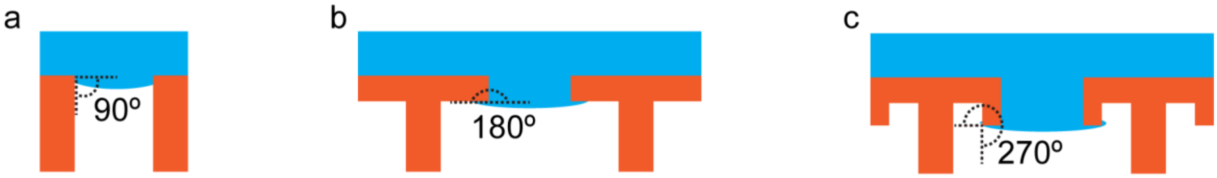

Figure S10. The edge angle of different fibrillar structures: a) simple pillars, b) T-shaped fiber structures, and c) double re-entrant fiber structures.

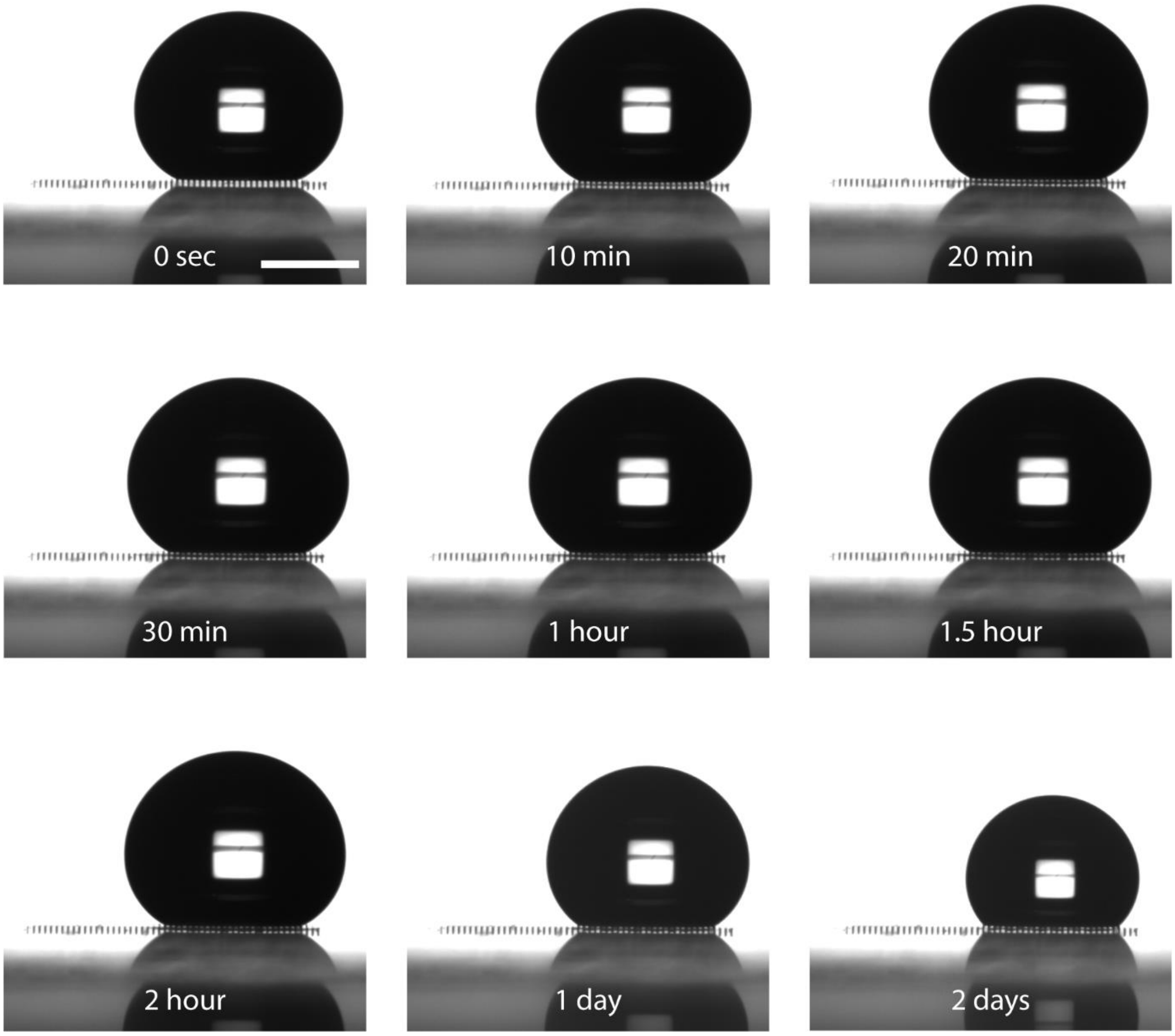

Figure S11. Liquid-repellency duration of the fabricated springtail- and gecko-inspired structures. The structures could repel a 5 µL ethylene glycol droplet for more than 2 days. After 2 days, the experiment was stopped. Scale bar: 1 mm.

Supplementary Movies

For each liquid repellency characterization in Movies S1-S4: The applied droplet volume was varied between ~2 µL and ~5 µL. The liquid dosing and aspiration speed was ~0.2 µL s^{-1}.

Movie S1: DI-water droplet on top of the springtail-gecko-inspired fibrils.

Movie S2: Ethylene glycol droplet on top of the springtail-gecko-inspired fibrils.

Movie S3: Ethanol droplet on top of the springtail-gecko-inspired fibrils.

Movie S4: Perfluorooctane droplet on top of the springtail-gecko-inspired fibrils.

Movie S5: Adhesion test sample video with a liquid droplet on the fabricated springtail-gecko-inspired fibril patch. The approaching and retraction speed was 25 µm s^{-1}. The relaxation time after preloading was 10 s. The preload was 6 mN. The contact surface was a hemispherical smooth *hydrophobic* glass indenter. The volume of the liquid droplet was 5 µL.

Movie S6: Adhesion test sample video with a liquid droplet on the fabricated springtail-gecko-inspired fibril patch. The approaching and retraction speed was 25 µm s^{-1}. The relaxation time after preloading was 10 s. The preload was 6 mN. The contact surface was a hemispherical smooth *hydrophilic* glass indenter. The volume of the liquid droplet was 5 µL.

Movie S7: Liquid-repellency duration demonstration of the fabricated springtail-gecko-inspired fibrils for DI-water. The volume of the droplet was 5 µL. The liquid-repellency duration was almost the same time with the evaporation time of the droplet.

References:

[1] A. Cassie, S. Baxter, *Trans. Faraday Soc.* **1944**, *40*, 546.

[2] T. Liu, C. J. Kim, *Science.* **2014**, *346*, 1096.

[3] X. Liu, H. Gu, M. Wang, X. Du, B. Gao, A. Elbaz, L. Sun, J. Liao, P. Xiao, Z. Gu, *Adv. Mater.* **2018**, *30*, DOI 10.1002/adma.201800103.
